# Clinical Outcomes Associated with Home Mechanical Ventilation: A Systematic Review

**DOI:** 10.1155/2016/6547180

**Published:** 2016-04-28

**Authors:** Erika J. MacIntyre, Leyla Asadi, Doug A. Mckim, Sean M. Bagshaw

**Affiliations:** ^1^Division of Critical Care Medicine, Faculty of Medicine and Dentistry, University of Alberta, Edmonton, AB, Canada T6G 2B7; ^2^Department of Medicine, Faculty of Medicine and Dentistry, University of Alberta, Edmonton, AB, Canada T6G 2R3; ^3^Division of Respirology and Respiratory Rehabilitation Services, Faculty of Medicine and Dentistry, University of Ottawa, Ottawa, ON, Canada T6G 2R3

## Abstract

*Background*. The prevalence of patients supported with home mechanical ventilation (HMV) for chronic respiratory failure has increased. However, the clinical outcomes associated with HMV are largely unknown.* Methods*. We performed a systematic review of studies evaluating patients receiving HMV for indications other than obstructive lung disease, reporting at least one clinically relevant outcome including health-related quality of life (HRQL) measured by validated tools; hospitalization requirements; caregiver burden; and health service utilization. We searched MEDLINE, EMBASE, CINAHL, the Cochrane library, clinical trial registries, proceedings from selected scientific meetings, and bibliographies of retrieved citations.* Results*. We included 1 randomized control trial (RCT) and 25 observational studies of mixed methodological quality involving 4425 patients; neuromuscular disorders (NMD) (*n* = 1687); restrictive thoracic diseases (RTD) (*n* = 481); obesity hypoventilation syndrome (OHS) (*n* = 293); and others (*n* = 748). HRQL was generally described as good for HMV users. Mental rather than physical HRQL domains were rated higher, particularly where physical assessment was limited. Hospitalization rates and days in hospital appear to decrease with implementation of HMV. Caregiver burden associated with HMV was generally high; however, it is poorly described.* Conclusion*. HRQL and need for hospitalization may improve after establishment of HMV. These inferences are based on relatively few studies of marked heterogeneity and variable quality.

## 1. Introduction

Home mechanical ventilation (HMV) is a viable and effective treatment strategy for patients with chronic respiratory failure and has been associated with a survival benefit [1–3]. Provision of mechanical ventilation outside of an institution has been in effect for more than 50 years, originating during the polio epidemic [4]. In recent decades, the prevalence has increased, with the expanded utilization of HMV for a broad range of neuromuscular disorders (NMD), restrictive thoracic diseases (RTD), and obesity hypoventilation syndrome (OHS). The Eurovent study estimated the prevalence of HMV users at 6.6 per 100,000 population, excluding OHS [1]. In comparison, recent Canadian data has determined that 10.9 per 100,000 population receive HMV [5]. The use of noninvasive ventilation (NIV) in stable chronic obstructive pulmonary disease (COPD) is an area of controversy. This group accounts for approximately one-third of users in Europe; however, given insufficient and often conflicting data current, Canadian guidelines do not support widespread usage in this population.

Health related quality of life (HRQL), constrained hospital resources, and increasing demands for prolonged mechanical ventilation have motivated providers, patients, and families to create a sustainable home environment for ventilatory assisted individuals (VAIs). While clinical practice guidelines to standardize the management of HMV have been developed, their implementation is uncertain [4]. This likely relates in part to variability in resource availability, inconsistent follow-up, clinician preference, and limited available scientific evidence [5].

While survival is indeed an important long-term outcome, use of HMV should also focus on additional patient-centered and system-level outcomes including HRQL, hospitalization needs, health resource utilization, and the role of caregivers. We therefore conducted a systematic review of the literature to examine all available studies evaluating at least one clinically relevant patient-centered and health resource utilization outcome in patients receiving HMV.

## 2. Methods

### 2.1. Search Strategy

A comprehensive search of MEDLINE, EMBASE, CINAHL, and the Cochrane library (1996 to August 2013) was performed with an experienced librarian (see Supplementary Methods in Supplementary Material available online at http://dx.doi.org/10.1155/2016/6547180). Three comprehensive search themes were derived and combined using the Boolean operator “AND”. The first theme included keyword/MESH headings related to disease states associated with chronic respiratory failure (CRF). The second theme was treatment related and included terms pertaining to all forms of mechanical ventilation. The third theme identified the setting of interest, this being a noninstitutional home environment. There were no restrictions on study design, language, or patient age. Studies published prior to 1996 were excluded to reflect important advances in home ventilator technology and patient management. In addition, we searched the abstracts from key scientific proceedings (CHEST, American Thoracic Society and European Respiratory Society, 2010–2013), the bibliographies of all retrieved articles and recent reviews, and clinical trials registries. Lastly content experts were contacted to locate additional sources of unpublished data that may be appropriate to our evidence synthesis.

### 2.2. Study Selection

Two trained reviewers (Erika J. MacIntyre and Leyla Asadi) independently conducted an initial eligibility screen of all retrieved titles and abstracts. Studies were selected for full text review if they met the following criteria: (1) study design (original research, other than case reports); (2) population (cohorts with CRF; studies examining COPD exclusively were not included as the use of HMV in stable COPD is currently not recommended in Canada); (3) intervention (invasive tracheostomy ventilation (TV) or NIV); (4) setting (noninstitutionalized residence); and (5) outcomes (patient- and/or family-centered outcomes other than survival and measures of health resource utilization; physiologic measures including blood gases and pulmonary function were not considered patient-centered; initial screening was broad and selection subsequently narrowed to our specific outcomes of interest).

### 2.3. Data Collection

Full text review, data extraction, and methodological quality scoring were independently performed by the same two reviewers on standardized data collection forms for studies that met inclusion criteria. Disagreements between reviewers were resolved through discussion and if consensus could not be achieved, discrepancies were resolved by a third reviewer (S.M.B.).

### 2.4. Methodological Quality Assessment

Methodological quality was scored using a modified version of the Downs and Black checklist (see Supplementary Methods) [31]. The original checklist contains 27 items; however, 10 items pertaining to interventional trials were excluded as only a single interventional trial was identified. In addition, the question of power was changed to whether the study commented on power and disclosure/conflict of interests was added. In the end, studies were scored by the following subscales: reporting [7], external validity [2], bias [5], confounding [3], and disclosure [1] for a total score range of 0 (poor quality) to 19 (rigorous).

### 2.5. Outcomes

All patient- and family-centered outcomes and health resource outcomes for this systematic review were determined a priori. Our primary outcomes included health related quality of life (HRQL) measured quantitatively by a validated HRQL assessment tool and hospitalization rates including number of admissions and days in hospital. Secondary outcomes consisted of family caregiver (FCG) burden defined by use of standardized or validated questionnaires, HRQL assessment tools or structured interviews grouped by domain, and health service utilization (including any interaction with a physician, allied health professional, or clinical test). Cost implications to either the system or families, sleep quality assessed by quantitative measures, and incidence of decannulation were also considered important secondary outcomes.

## 3. Results

### 3.1. Study Characteristics

**Figure 1:**
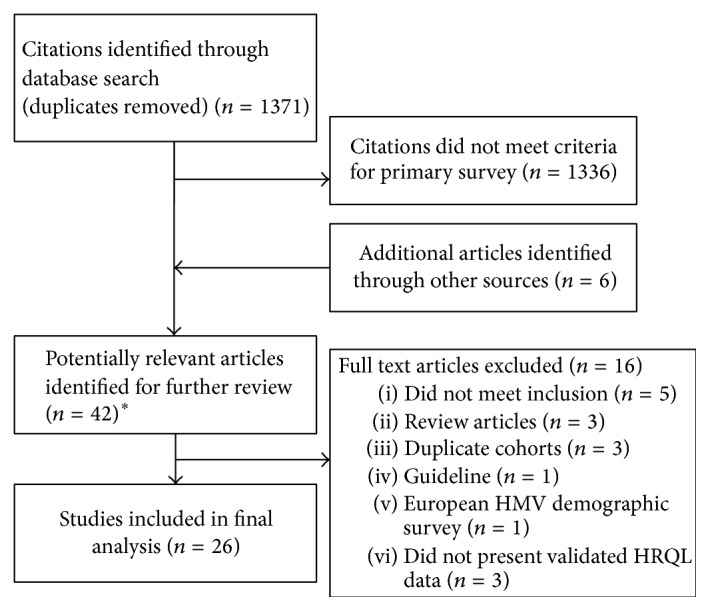
Outline of study selection process. HMV, home mechanical ventilation; HRQL, health related quality of life. ^*∗*^4 German language studies.

The database search generated 1371 citations, of which 36 met inclusion criteria. An additional 6 articles, identified through other sources, were included in the full text review for a total of 42 articles. German language articles (*n* = 4) were screened for content by one reviewer (Erika J. MacIntyre) and a professional translator (Regina Landeck). Reasons for exclusion are shown in Figure 1.

After screening, we included 1 randomized control trial (RCT) [3] and 25 observational studies (before and after, *n* = 10 [6–15]; cohort, *n* = 3 [16–18]; cross-sectional surveys, *n* = 11 [19–22, 24–29, 32]; descriptive, *n* = 1 [30]) (Table 1).

### 3.2. Patient Characteristics

A total of 4425 patients were studied. There were three broad disease states contributing to CRF and establishment of HMV: NMD (patients, *n* = 1697); RTD (*n* = 481); OHS (*n* = 293). One study did not provide the number of patients in each disease category (*n* = 1211). The remainder were classified as “other,” defined as a mixture of patients including many with COPD (*n* = 748) (Table 2). NIV was used in the majority (85%) of patients (*n* = 3682).

### 3.3. Methodological Quality Assessment

Overall quality was variable (Table 1). Scores ranged from 4 for the descriptive study to 18 for the RCT out of 19 (see Supplementary Results). Median scores were 14 for before and after studies (range 8–17), 17 for cohorts [13–16], and 10 for cross-sectional surveys [5–14, 31]. The greatest deficiencies across studies were in the categories of confounding (median score 1 out of 3) and disclosure (reported in only 6 studies).

### 3.4. Health Related Quality of Life

Eleven studies provided data on HRQL using one or more general or disease-specific tools, including the Short Form-36 (SF-36), Sickness Impact Profile (SIP), Severe Respiratory Insufficiency (SRI) questionnaire, Profile of Mood States (POEMS), Munich Quality of Life Dimensions (MLDL), Health Index (HI), Sense of Coherence (SOC) scale, Saint George Respiratory Questionnaire (SGRQ), and the Chronic Respiratory Disease Questionnaire (CRQ) (see Supplementary Results) [3, 6–8, 11–13, 17, 22, 25, 26].

HRQL was generally described as good. Five studies examined HRQL before and after HMV using the SF-36 [3, 6, 7, 12, 13].  Table 3 presents statistically significant changes by item.

Improved HRQL was more consistent across the mental compared to the physical domains. With respect to mental capacity scores, every study examining OHS or RTD reported improvement in at least one domain or summary score following the establishment of HMV. There was no evidence of a clinically important deterioration in any group. Physical component scores were more heterogeneous. In NMD, one study found deterioration in the domain of physical functioning and improvement only seen in the item of general health in a single study; otherwise there was no change. The majority of the studies examining the remaining disease states reported benefit in at least one physical domain and/or overall physical component score.

The remaining 6 studies were of mixed design and applied different HRQL tools [7, 11, 17, 22, 25]. Several of these compared HRQL between disease states with conflicting results [7]. Using the SIP instrument, Markström et al. found significant functional impairment (score of >10%) in NMD (*n* = 49) compared to no clinical dysfunction in RTD (score of <5%) (*n* = 19), while Dellborg et al. noted comparable improvement in both NMD (*n* = 12) and RTD (*n* = 21) [8, 26].

### 3.5. Hospitalization

Nine studies (36%) examined hospitalization rates following the institution of HMV (patients, *n* = 1528) [6, 7, 9–12, 14, 20, 32]. Results are presented in Table 4, omitting one study which did not present admission rates immediately following HMV establishment [20]. In general, the initial titration took place in hospital. Hospitalization rates and days in hospital overall appear to be low and to decrease in subsequent years across studies and disease states following the implementation of HMV. Reasons for admission were related predominately to cardiac or respiratory related problems where specified [7, 10, 11, 14]. Admissions for ventilator setting adjustments or equipment servicing were infrequent and generally not considered. Interpretation of study findings is limited and could not be quantitatively pooled due to heterogeneity across diseases and reporting methods [6, 11, 32]. One study found hospitalization rates to be one of the strongest predictors of impaired HRQL [22].

### 3.6. Secondary Outcomes

Family caregiver (FCG) characteristics are presented in Table 5 (studies, *n* = 8; caregivers, *n* = 555) [18, 19, 21, 24, 25, 28, 29]. FCGs of TV users were predominantly examined (NIV use 45% versus 85% when all studies were examined). FCGs were younger than HMV users, predominantly female (76%) and of spousal (54%) or parental (31%) relation. Fewer than half were actively employed and many described having to quit work or reduce work hours to enable care for VAIs. Given the choice, 80% of FCGs would choose HMV again for their loved ones. This number is higher (88%) in studies that examined caregivers of NMD and RTD patients only compared to the study that included caregivers of a mixed cohort (42%) [19, 25, 27, 29]. Parental caregivers also appeared to have a greater sense of satisfaction with their decision [18, 19, 27]. Some attempted to quantify burden using a variety of tools including the Family Burden Questionnaire, the Zarit interview, and the caregiver burden inventory. All studies highlighted burden in the domains of financial strain, negative impact on employment, and insufficient time for oneself and for personal relationships [19, 21, 24].

Health resource use and the economic impact of HMV, beyond hospitalization, were assessed in 7 studies [16, 20, 23, 25, 28–30, 32]. Access to medical experts, phone calls to a unit specializing in HMV, ventilator servicing, home visits by technicians or nursing staff, and education were identified as important resources [16, 20, 32]. Cost was high and home care appeared to be the largest expense. Despite most patients having some level of health insurance coverage, many families suffered out-of-pocket expenses either in the form of lost wages or in order to privately fund home care, medications, or equipment. Crude cost estimates are provided in Table 6. Despite this, Moss et al. observed significantly less cost with home compared to institutionalized VAIs [29].

All studies presenting data on sleep quality documented improvements in either overall sleep quality or sleep related symptoms including morning headaches, nocturnal dyspnea, and daytime somnolence with HMV [3, 8, 10]. No study discussed decannulation in an ambulatory setting.

## 4. Discussion 

In our scoping review we identified 26 unique studies reporting data on at least one patient-centered outcome other than survival in 4425 HMV users.

### 4.1. Summary of Major Findings

We found that HMV generally had a favorable impact on HRQL. Not surprisingly, improvement was more prominent and consistent for mental domains compared with physical domains across HRQL measures, particularly in those with NMD. We found HMV may be associated with an initially low and a subsequently reduced rate of hospitalization and days in hospital following implementation. While poorly described, FCG burden appears quite high. This likely relates in part to the financial strain associated with keeping VAIs in a home environment and may contribute to the unwillingness of some caregivers to choose HMV for their loved one again if given a second opportunity.

### 4.2. Context with Previous Studies

We believe our findings require thoughtful interpretation given that they are based on relatively few studies of mixed design and methodological quality describing small cohorts of heterogeneous patients. In general, pooled quantitative analyses could not be performed due to heterogeneity across studies, particularly in terms of the measures of HRQL, hospitalization requirements and caregiver burden that were applied. In addition, our findings are also likely confounded by concomitant cointerventions associated with entry into a multidisciplinary HMV program, such as improved medical attention, education, airway clearance strategies, home care, and provision of mobility aids [4, 33, 34]. With the exception of one study, HMV was offered to all individuals with CRF and as such, there were no proper controls for comparison. However, the natural history of many of these chronic incurable diseases includes progressive decline in health status with an expected increased health resource use which was not seen.

For NMD, there are plausible reasons for the lack of improvement in HRQL association with initiation of HMV. First, many NMD are progressive and can be associated with rapid clinical deterioration. In ALS, HRQL is generally worse in those with bulbar muscle dysfunction and HMV may be of limited benefit [3]. Second, ten physical component items in the SF-36 assess the ability to perform physical tasks, which may not be possible for patients with advanced NMD. The SRI instrument may address some of these shortcomings and appears to be a more sensitive HRQL assessment tool in HMV users [35].

We also examined several clinically important secondary outcomes. Family care givers were often younger, female, and of spousal or parental relation. Most FCGs were caring for loved ones receiving TV, suggesting a greater burden associated with invasive ventilation. Importantly, nearly one-fifth of all FCGs would not choose the option of HMV again, if given the opportunity, suggesting that the perceived burden is unmanageably high for many. It would seem that there are differences in caregiver perceptions of burden across disease states with burden being the least in caregivers of patients with NMD and RTD, particularly in the case of parents, slightly higher in ALS where the FCG is more often a spouse, and highest in caregivers of mixed cohorts. Moreover, these studies reinforce that FCG burden for HMV patients is poorly evaluated and understood.

There is limited data on the health economic implications of HMV; however, we can make several observations. There appears to be wide variation in the policies, procedures, and patterns of practice around the assessment, initiation, and maintenance of patients on HMV across health jurisdictions. Ambulatory service provision is necessary to maintain patients in a home environment, but this is currently not standardized. Some centers have specialized multidisciplinary home ventilation units that follow all VAIs, whereas in others follow-up can be infrequent and may not even involve a physician. The costs of maintaining VAIs at home are high; however, this is likely significantly less than the cost of long-term institutionalization. Many FCG suffered out-of pocket expenses and financial strain was highlighted in all 8 studies where caregiver burden was examined. This would strongly suggest that the current level of home support is inadequate.

No studies reported on decannulation. This is likely due to the following: (1) NIV use became the preferred first-line method of HMV by patients and providers for which weaning may be less relevant compared with invasive TV. Moreover, elective “ramping” of up to 24 hours of NIV may often obviate the need for invasive TV; (2) HMV patients are referred from chronic ventilation units where patients may have already failed to wean; and (3) in many patients, respiratory recovery is unlikely.

### 4.3. Strengths and Limitations

A prior systematic review aimed to evaluate the impact of HMV on hospitalization and sleep quality in NMD and RTD; however, the paucity of data limited the capacity for clear inferences [2]. In addition, a recent systematic review evaluated the impact of NIV on patient reported outcomes in a similar population. Their conclusions regarding HRQL were largely coherent with our findings; however, there was no assessment of the impact of NIV on hospitalizations, health resource implications, or FCG burden [36]. Our review has overcome some of these limitations by using a broader search strategy [37]. We believe our findings, based on a larger body of literature, better represent the impact of HMV on patient-centered outcomes beyond survival.

Our study also has notable limitations. Firstly, small sample size, heterogenous study design, and variable methodological quality weaken the strength of inferences from our study. Secondly, our outcomes may have been influenced by methods of ventilation and compliance. Compliance is generally considered use for >4 hours/night, 5 nights/week, and while this was an item in our assessment of methodological quality, duration and usage were not fully taken into consideration. Thirdly, many of our outcomes were prone to bias, particularly survivorship bias. Fourthly, not all relevant patient-centered outcomes were considered and we acknowledge that not all outcomes are of equal value to patients and/or FCGs. Fifthly, there is heterogeneity within disease categories and our findings may not be applicable across all specific disease states. Lastly, as previously stated, concomitant cointerventions associated with entry into a multidisciplinary program confound and may exaggerate the beneficial effects of HMV.

### 4.4. Clinical and Health Policy Implications

The utilization of prolonged mechanical ventilation in CRF secondary to NMD, RTD, and OHS is increasing and likely represents a significant escalation in the complexity and cost of home care. Uncertainty surrounding optimal HMV service provision on patient-centered outcomes is an important knowledge gap and lack of evidence undoubtedly contributes to wide variation in clinical practice, including patterns of home ventilation initiation and titration, which may incur considerable costs to the health system. A large RCT evaluating HMV versus a nonventilated control would be challenging and perhaps unethical. We therefore will need to rely on high quality observational data and randomized trials of selected processes of care to further our understanding of the ideal method of HMV to optimize patient-centered outcomes. Then resources can be appropriately allocated by policy makers and providers along with a greater homogenization of standard practices to ensure high quality care is delivered to patients and their families.

## 5. Conclusion

In summary, our systematic review suggests that HMV likely provides quality of life benefit and reduced hospitalizations in patients with CRF secondary to NMD, RTD, and OHS. However, small sample sizes, heterogeneity in study design, and variable methodological quality weaken these inferences. With the preferential and proactive implementation of noninvasive over invasive ventilation, the utilization of HMV is likely to expand considerably. Future investigations are clearly needed to better understand the optimal methods for providing care for HMV patients, along with associated caregiver concerns and health economic implications.

## Supplementary Material

Supplementary material contains the literature search strategy, the modified Down's and Black study quality assessment, a detailed list of disease states and a brief explanation of the health related quality of life and caregiver assessment tools that were used.

## Figures and Tables

**Table 1:** Study characteristics by study design and year of publication.

Study	Year	Study design	Location	*n*	f/u (mo)	Quality^*∗*^
Bourke et al. [3]	2006	RCT	SC UK	41	12	18
Tsolaki et al. [6]	2011	BA	SC Greece	91	24	15
Windisch et al. [7]	2008	BA	MC Germany	85	12	16
Dellborg et al. [8]	2008	BA	MC Sweden	35^†^ 11^‡^	9^†^ 8 y^‡^	13
Farrero et al. [9]	2006	BA	SC Spain	43	36	16
Gonzalez et al. [10]	2003	BA	SC Spain	16	3y	8
Janssens et al. [11]	2003	BA	MC Switzerland	211	1–88	15
Nauffal et al. [12]	2002	BA	SC Spain	62	18	12
Hein et al. [13]	1999	BA	SC Germany	27	2–30	13
Bach et al. [14]	1998	BA	MC USA	684	V	13
Janssens et al. [15]	1998	BA	SC Switzerland	6	17–55	14
Chatwin et al. [16]	2010	C	SC UK	1211	6	14
Budweiser et al. [17]	2007	C	SC Germany	231	29	17
Marchese et al. [18]	2007	C	SC Italy	77	V	17
Evans et al. [19]	2012	CSS	SC Canada	12	NA	12
Chang et al. [20]	2010	CSS	SC New Zealand	45	NA	10
Fernández-Álvarez et al. [21]	2008	CSS	SC Italy	66	NA	10
López-Campos et al. [22]	2008	CSS	MC Spain	115	NA	15
Vitacca et al. [23]	2007	CSS	MC Italy, Spain	792	NA	13
Tsara et al. [24]	2004	CSS	SC Greece	50	NA	10
Kaub-Wittemer et al. [25]	2003	CSS	SC Germany	53	NA	5
Markström et al. [26]	2002	CSS	SC Sweden	91	NA	9
van Kesteren et al. [27]	2001	CSS	SC Netherlands	38	NA	8
Sevick and Bradham [28]	1997	CSS	MC USA	277	NA	9
Moss et al. [29]	1996	CSS	MC USA	50^§^	NA	6
Bötel et al. [30]	1997	D	SC Germany	16	NA	4

*n*, clinically evaluable population; RCT, randomized control trial; BA, before and after; C, cohort; CSS, cross-sectional survey; D, descriptive; SC, single center; MC, multicenter; NA, not applicable or not assessed; V, variable.

^*∗*^Modified Downs and Black score out of 19. ^†^Cohort who survived to 9 mo. ^‡^Cohort who survived to 8 y. ^§^16 institutionalized.

**Table 2:** Patient characteristics.

Study	Age (mean)		Male (*n*)	*n*	NMD (*n*)	RTD (*n*)	OHS (*n*)	Other^‡‡^ (*n*)	NIV (*n*)
Bourke et al. [3]	63^*∗*^		24	41	41	NA	NA	NA	22
Tsolaki et al. [6]	65^†^		61	91	11	17	28	35	91
Windisch et al. [7]	59^†^		49	85	17	29	9	27	85
Dellborg et al. [8]	63^‡^		15^‡^	35^‡^	12^‡^	21^‡^	NA	2^‡^	35
	58^§^		4^§^	11^§^	7^§^	4^§^		0^§^	
Farrero et al. [9]	77^‖^		20	43	9	26	8	0	43
Gonzalez et al. [10]	57		NR	16	NA	16	NA	NA	16
Janssens et al. [11]	63^†^		NR	211	40	42	71	58	211
Nauffal et al. [12]	50^†^		33	62	27	35	NA	NA	62
Hein et al. [13]	56		16	27	8	8	NA	11	NR
Bach et al. [14]	NR		NR	684	650	19	3	12	636
Janssens et al. [15]	79		2	6	0	4	1	1	6
Chatwin et al. [16]	46		642	1211	NR	NR	NR	NR	1199
Budweiser et al. [17]	63		146	231	15	49	69	98	226
Marchese et al. [18]	58		54	77	41	8	3	25	0
Evans et al. [19]	45		NR	12	12	NA	NA	NA	0
Chang et al. [20]	55		24	45	7	6	27	3	39
Fernández-Álvarez et al. [21]	61		34	66	10	11	27	18	64
López-Campos et al. [22]	62		58	115	18	45	37	15	115
Vitacca et al. [23]	67		562	792	375	128	NA	289	634
Tsara et al. [24]	61^¶^		NR	50	15	0	6	29	44
Kaub-Wittemer et al. [25]	61		42	53	53	NA	NA	NA	32
Markström et al. [26]	59		40	91	49	13	0	29	55
van Kesteren et al. [27]	34		24	38	34	4	NA	NA	12
Sevick and Bradham [28]	45		NR	277	177	0	4	96	48
Moss et al. [29]	59		34	50	50	NA	NA	NA	7
Bötel et al. [30]	NA		NA	16	16	NA	NA	NA	NR

Aggregate data	*n*	60	1880	4425	1687	481^*∗∗*^	293	748	3682
	%^††^	NA	59	100	53	15^*∗∗*^	9	23	85

*n*, clinically evaluable population; NIV, noninvasive ventilation; NR, not reported.

^*∗*^Age >75 exclusion criteria. ^†^Value based on mean age data provided for each disease state. ^‡^Cohort who survived to 9 mo. ^§^Cohort who survived to 8 y. ^‖^Age >75 inclusion criteria. ^¶^Median. ^*∗∗*^Several studies grouped RTD with NMD; ^††^% aggregate proportion was based on total clinically evaluable population where both the numerator and denominator were available within the study. ^‡‡^Including lung disease.

**Table 3:** Change in SF-36 domains at 12 mo with home ventilation compared to baseline across studies and disease states.

Disease state	Study	*n*	Physical capacity					Mental capacity				
			PF	RP	BP	GH	PCS	Vi	SF	RE	MH	MCS
RTD	Windisch et al. [7]	29	NC	NC	NC	NC	NC	+	+	+	+	NC
	Hein et al. [13]	8	NC	NC	NC	NC	NR	+	NC	NC	+	NR
	Nauffal et al. [12]	35	NC	+	NC	NC	NR	NC	+	+	NC	NR
	Tsolaki et al. [6]	17	NR	NR	NR	NR	+	NR	NR	NR	NR	+

NMD	Windisch et al. [7]	17	NC	NC	NC	NC	NC	NC	+	NC	NC	NC
	Hein et al. [13]	8	NC	NC	NC	NC	NR	NC	NC	NC	+	NR
	Nauffal et al. [12]	27	—	NC	NC	NC	NR	NC	NC	NC	NC	NR
	Bourke et al. [3]^*∗*^	41	NC	NC	NC	+	NC	+	NC	NC	+	+
	Bourke et al. [3]^†^	20	NC	NC	NC	+	NC	+	+	+	+	+
	Tsolaki et al. [6]	11	NR	NR	NR	NR	NC	NR	NR	NR	NR	NC

OHS	Windisch et al. [7]	9	+	+	NC	+	+	+	NC	+	+	+
	Tsolaki et al. [6]	28	NR	NR	NR	NR	+	NR	NR	NR	NR	+

BP, bodily pain; GH, general health; MH, mental health; NC, no change; NMD, neuromuscular disorders; NR, not reported; OHS, obesity hypoventilation syndrome; PF, physical function; RE, role emotional; RP, role physical; RTD, restrictive thoracic diseases; SF, social functioning; Vi, vitality; +, statistically significant improvement; —, statistically significant deterioration.

^*∗*^Amyotrophic lateral sclerosis (ALS) only; ^†^ALS with preserved bulbar function. There were no improvements within any of the domains in the poor bulbar function subgroup.

**Table 4:** Hospitalization rates before and after HMV.

Study	Disease state	*n*	Before^*∗*^	After^†^
			#admissions/y	#admissions/y
Windisch et al. [7]	NMD, RTD, COPD, OHS	85	NR	0.1
Nauffal et al. [12]	NMD	27	1.1 (1.2)	0.3 (1.2)
	RTD	35	1.2 (1.8)	0.8 (1.2)
Farrero et al. [9]	NMD, RTD, OHS	43	2.2 (2.4)	0.5 (0.6)
Bach et al. [14]	NMD, RTD	654	1.5 (2.5)^‡^	<0.6^§^
Vitacca et al. [23]	NMD	375	NR	0.5 (0.4)^‖^
	RTD	128	NR	0.8 (0.5)^‖^

			d in hospital/pt/y	d in hospital/pt/y

Tsolaki et al. [6]	NMD	11	NR	2.4 (NR)
	RTD	17	NR	0
	OHS	28	NR	3.8 (5.7)
Janssens et al. [11, 15]	NMD, RTD	77	22 (2)	17 (4)
	OHS	32	26 (4)	17 (5)
Gonzalez et al. [10]	RTD	16	10.9 (13.3)	0

d, day; pt, patient; NMD, neuromuscular disorders; NR, not reported; OHS, obesity hypoventilation syndrome; RTD, restrictive thoracic diseases; y, year.

^*∗*^Mean (SD) based on 3-month minimum duration. ^†^Mean (SD) based on first year of follow-up; ^‡^204 high risk patients (defined as beginning from the first episode of pneumonia or hospitalization for respiratory failure until treatment with oxygen or mechanical ventilation). ^§^Aggregate data was not available and was instead reported by method of ventilation. For all methods hospitalization rates were <0.6. ^‖^Median (SD).

**Table 5:** Primary caregiver characteristics.

Study	*n*	Age (mean)	Male (*n*, %)	Spouse (*n*, %)	Parent (*n*, %)	Child (*n*, %)	Employed (*n*, %)	Would choose HMV again (*n*, %)	NIV (*n*, %)
Studies included caregivers of patients with NMD and RTD only									
Evans et al. [19]	12	55	3 (25)	6 (50)	5 (42)	1 (8)	NR	NR	0
Kaub-Wittemer et al. [25]^*∗*^	52	56	10 (19)	51 (98)	0	1 (2)	34 (65)^¶^	46 (88)^‡‡^	32 (62)
van Kesteren et al. [27]	31^‡^	NR	NR	12 (39)	19 (61)	0	NA	29 (94)	23 (74)
Moss et al. [29]^*∗*^	36	NR	10	NR	NR	NR	NA	30 (83)	7 (19)
Aggregate data (*n*, %^†^)	131	56	23 (23)	69 (73)	24 (25)	2 (2)	NA	105 (88)	62 (47)

Studies included caregivers of patients with NMD, RTD, and other conditions									
Marchese et al. [18]	77	NR	15 (19)	55 (71)	18 (23)	3 (4)	NR	42 (55)^§§^	77 (100)
Fernández-Álvarez et al. [21]	20^§^	51	5 (25)	NR	NR	NR	14 (70)	NR	19 (95)
Tsara et al. [24]	50	48^‖^	NR	21 (42)	NR	20 (40)	24 (48)^*∗∗*^	NR	44 (88)
Sevick and Bradham [28]	277	53	69 (25)	127 (46)	111 (40)	NR	119 (43)^††^	NR	48 (96)
Aggregate data (*n*%^†^)	424	51	89 (24)	203 (54)	129 (34)	20 (5)	157 (45)	42 (55)	188 (44)

All studies examining caregiver burden									
Aggregate data total (*n*, %^†^)	555	53	112 (24)	272 (55)	153 (31)	25 (5)	191 (48)	157 (80)	250 (45)

HMV, home mechanical ventilation; NA, not assessed; NR, not reported.

^*∗*^Caregivers of amyotrophic lateral sclerosis (ALS) patients exclusively; ^†^% aggregate proportion was based on total clinically evaluable population where both the numerator and denominator were available within the study. ^‡^For 16 patients both parents were interviewed; however only one was considered in the table. ^§^Caregivers for the subgroup of highly dependent patients (Katz index level C). ^‖^Median. ^¶^6 caregivers of NIV and 12 caregivers of TV users quit work to care for HMV user. This value is assuming that all caregivers were employed initially. ^*∗∗*^Several caregivers quit their job or had to ask for time off work; however this number is not reported;^ ††^179 had been employed. 60 stopped working; 40 decreased their hours; 16 changed jobs; and 3 increased hours. ^‡‡^31 caregivers of NIV users and 15 of TV users.

^§§^Including all parents.

**Table 6:** Crude cost estimates.

Study/disease state	Annual cost, mean and/or range^*∗*^			Cost coverage
	Home care	Home care + equipment	Out-of-pocket and/or lost wages	
Kaub-Wittemer et al. [25]/NMD	NR	NR	3,624–29,942^†^ 3,151–78,955^‡^	All were eligible for federally funded nursing care

Sevick and Bradham [28]/COPD, NMD/OHS	116,222	NR	14,412^§^ 0–545,449	NR

Moss et al. [29]/ALS		208,738	15,829 0–367,925	91% privately insured Insurance covered >94% of expenses for 64% of patients

Bötel et al. [30]/SCI	552,260	732,250	NR	NR

ALS, amyotrophic lateral sclerosis; COPD, chronic obstructive pulmonary disorders; NMD, neuromuscular disorders; NR, not reported; OHS, obesity hypoventilation syndrome; SCI, spinal cord injury. ^*∗*^Converted to US currency from publication date to September 29, 2014; ^†^4 of 32 NIV users reported out-of-pocket expenses; ^‡^5 of 21 TV users reported out-of-pocket expenses; ^§^mean change to gross salary.

## Abbreviations

HMV: Home mechanical ventilation
NIV: Noninvasive ventilation
TV: Tracheostomy ventilation
CRF: Chronic respiratory failure
BPAP: Bilevel positive airway pressure
VAI: Ventilator assisted individual
COPD: Chronic obstructive pulmonary disease
NMD: Neuromuscular disorders
RTD: Restrictive thoracic disease
OHS: Obesity hypoventilation syndrome
HRQL: Health related quality of life
FCG: Family caregiver
SF-36: Short Form-36
SIP: Sickness Impact Profile
SRI: Severe Respiratory Insufficiency questionnaire
RCT: Randomized control trial.

## References

[1] Lloyd-Owen S. J., Donaldson G. C., Ambrosino N. (2005). Patterns of home mechanical ventilation use in Europe: results from the Eurovent survey. *European Respiratory Journal*.

[2] Annane D., Orlikowski D., Chevret S., Chevrolet J., Raphael J. C. (2009). Nocturnal mechanical ventilation for chronic hypoventilation in patients with neuromuscular and chest wall disorders (Review). *The Cochrane Library*.

[3] Bourke S. C., Tomlinson M., Williams T. L., Bullock R. E., Shaw P. J., Gibson G. J. (2006). Effects of non-invasive ventilation on survival and quality of life in patients with amyotrophic lateral sclerosis: a randomised controlled trial. *The Lancet Neurology*.

[4] McKim D. A., Road J., Avendano M. (2011). Home mechanical ventilation: a Canadian Thoracic Society clinical practice guideline. *Canadian Respiratory Journal*.

[5] Rose L., McKim D. A., Katz S. L. (2015). Home mechanical ventilation in Canada: a national survey. *Respiratory Care*.

[6] Tsolaki V., Pastaka C., Kostikas K. (2011). Noninvasive ventilation in chronic respiratory failure: effects on quality of life. *Respiration*.

[7] Windisch W., Barchfeld T., Freidel K. (2008). Impact of home mechanical ventilation on health-related quality of life. *European Respiratory Journal*.

[8] Dellborg C., Olofson J., Midgren B. (2008). Impact of home mechanical ventilation on health-related quality of life in patients with chronic alveolar hypoventilation: a prospective study. *The Clinical Respiratory Journal*.

[9] Farrero E., Prats E., Manresa F., Escarrabill J. (2007). Outcome of non-invasive domiciliary ventilation in elderly patients. *Respiratory Medicine*.

[10] Gonzalez C., Ferris G., Diaz J., Fontana I., Nuñez J., Marín J. (2003). Kyphoscoliotic ventilatory insufficiency: effects of long-term intermittent positive-pressure ventilation. *Chest*.

[11] Janssens J.-P., Derivaz S., Breitenstein E. (2003). Changing patterns in long-term noninvasive ventilation: a 7-year prospective study in the Geneva Lake Area. *Chest*.

[12] Nauffal D., Doménech R., Martínez García M. A., Compte L., Macián V., Perpiñá M. (2002). Noninvasive positive pressure home ventilation in restrictive disorders: outcome and impact on health-related quality of life. *Respiratory Medicine*.

[13] Hein H., Schucher B., Magnussen H. (1999). Quality of life of various patient groups during home mechanical ventilation. *Medizinische Klinik (Munich)*.

[14] Bach J. R., Rajaraman R., Ballanger F. (1998). Neuromuscular ventilatory insufficiency: effect of home mechanical ventilator use v oxygen therapy on pneumonia and hospitalization rates. *American Journal of Physical Medicine and Rehabilitation*.

[15] Janssens J.-P., Cicotti E., Fitting J.-W., Rochat T. (1998). Non-invasive home ventilation in patients over 75 years of age: tolerance, compliance, and impact on quality of life. *Respiratory Medicine*.

[16] Chatwin M., Heather S., Hanak A., Polkey M. I., Simonds A. K. (2010). Analysis of home support and ventilator malfunction in 1,211 ventilator-dependent patients. *European Respiratory Journal*.

[17] Budweiser S., Hitzl A. P., Jörres R. A., Schmidbauer K., Heinemann F., Pfeifer M. (2007). Health-related quality of life and long-term prognosis in chronic hypercapnic respiratory failure: a prospective survival analysis. *Respiratory Research*.

[18] Marchese S., Lo Coco D., Lo Coco A. (2008). Outcome and attitudes toward home tracheostomy ventilation of consecutive patients: a 10-year experience. *Respiratory Medicine*.

[19] Evans R., Catapano M. A., Brooks D., Goldstein R. S., Avendano M. (2012). Family caregivers perspectives on caring for ventilator-assisted individuals at home. *Canadian Respiratory Journal*.

[20] Chang A. Y., Marsh S., Smith N., Neill A. (2010). Long-term community non-invasive ventilation. *Internal Medicine Journal*.

[21] Fernández-Álvarez R., Rubinos-Cuadrado G., Cabrera-Lacalzada C., Galindo-Morales R., Gullón-Blanco J. A., González-Martín I. (2009). Home mechanical ventilation: dependency and burden of care in the home. *Archivos de Bronconeumologia*.

[22] López-Campos J. L., Failde I., Masa J. F. (2008). Factors related to quality of life in patients receiving home mechanical ventilation. *Respiratory Medicine*.

[23] Vitacca M., Assoni G., Pizzocaro P. (2006). A pilot study of nurse-led, home monitoring for patients with chronic respiratory failure and with mechanical ventilation assistance. *Journal of Telemedicine and Telecare*.

[24] Tsara V., Serasli E., Voutsas V., Lazarides V., Christaki P. (2006). Burden and coping strategies in families of patients under noninvasive home mechanical ventilation. *Respiration*.

[25] Kaub-Wittemer D., Von Steinbüchel N., Wasner M., Laier-Groeneveld G., Borasio G. D. (2003). Quality of life and psychosocial issues in ventilated patients with amyotrophic lateral sclerosis and their caregivers. *Journal of Pain and Symptom Management*.

[26] Markström A., Sundell K., Lysdahl M., Andersson G., Schedin U., Klang B. (2002). Quality-of-life evaluation of patients with neuromuscular and skeletal diseases treated with noninvasive and invasive home mechanical ventilation. *Chest*.

[27] van Kesteren R. G., Velthuis B., van Leyden L. W. (2001). Psychosocial problems arising from home ventilation. *American Journal of Physical Medicine and Rehabilitation*.

[28] Sevick M. A., Bradham D. D. (1997). Economic value of caregiver effort in maintaining long-term ventilator-assisted individuals at home. *Heart & Lung*.

[29] Moss A. H., Oppenheimer E. A., Casey P. (1996). Patients with amyotrophic lateral sclerosis receiving long-term mechanical ventilation: advance care planning and outcomes. *Chest*.

[30] Bötel U., Gläser E., Niedeggen A., Meindl R. (1997). The cost of ventilator-dependent spinal cord injuries-patients in the hospital and at home. *Spinal Cord*.

[31] Downs S. H., Black N. (1998). The feasibility of creating a checklist for the assessment of the methodological quality both of randomised and non-randomised studies of health care interventions. *Journal of Epidemiology and Community Health*.

[32] Vitacca M., Escarrabill J., Vianello A. (2007). Home mechanical ventilation patients: a retrospective survey to identify level of burden in real life. *Monaldi Archives for Chest Disease*.

[33] Bach J. R. (2002). Amyotrophic lateral sclerosis: prolongation of life by noninvasive respiratory aids. *Chest*.

[34] Gomez-Merino E., Bach J. R. (2002). Duchenne muscular dystrophy: prolongation of life by noninvasive ventilation and mechanically assisted coughing. *American Journal of Physical Medicine and Rehabilitation*.

[35] Windisch W., Freidel K., Schucher B. (2003). The Severe Respiratory Insufficiency (SRI) Questionnaire A specific measure of health-related quality of life in patients receiving home mechanical ventilation. *Journal of Clinical Epidemiology*.

[36] Hannan L. M., Dominelli G. S., Chen Y.-W., Darlene Reid W., Road J. (2014). Systematic review of non-invasive positive pressure ventilation for chronic respiratory failure. *Respiratory Medicine*.

[37] Stroup D. F., Berlin J. A., Morton S. C. (2000). Meta-analysis of observational studies in epidemiology: a proposal for reporting. Meta-analysis of Observational Studies in Epidemiology (MOOSE) group. *The Journal of the American Medical Association*.

