# Supplementary material for: Clinical Outcomes Associated with Home Mechanical Ventilation: A Systematic Review
Source: Can Respir J. 2016 Apr 28;2016:6547180. doi: 10.1155/2016/6547180 (PMC4904519; doi:10.1155/2016/6547180)
Supplement: Supplementary file 1 — Supplementary material contains the literature search strategy, the modified Down's and Black study quality assessment, a detailed list of disease states and a brief explanation of the health related quality of life and caregiver assessment tools that were used. [file 6547180.f1.pdf]

SUPPLEMENTARY FILE

SUPPLEMENTARY METHODS

Our scoping review adhered to the MOOSE statement for reporting of observational studies where possible[1]. We referred to the general guidelines of the Cochrane Handbook for Systematic Reviews of Interventions[2].

Literature search strategy

Ovid MEDLINE(R) In-Process & Other Non-Indexed Citations, Ovid MEDLINE(R) Daily and Ovid MEDLINE(R) 1946 to Present
Searched August 19, 2013 (638 results)

1. Lung Diseases/
2. Lung Diseases, Obstructive/
3. Pulmonary Disease, Chronic Obstructive/
4. neuromuscular diseases/ or exp motor neuron disease/ or exp muscular atrophy, spinal/ or muscular diseases/ or muscular disorders, atrophic/ or exp myopathies, structural, congenital/ or exp myositis/ or exp myotonic disorders/
5. respiratory insufficiency/ or exp hypoventilation/
6. Respiration Disorders/
7. Hypercapnia/
8. Spinal Cord Injuries/
9. kyphosis/ or scoliosis/
10. (COPD or respiratory insufficiency or respiratory failure or breathing failure or breathing difficult* or respiratory muscle weakness or ("pulmonary function" and failure) or pulmonary disease or amyotrophic lateral sclerosis or ALS or OHS or hypoventilation or spinal cord injur* or SCI or muscular dystroph* or Kyphoscoliosis or Postpoliomyelitis Syndrome or post-polio syndrome or post polio syndrome or polio syndrome or post-poliomyelitis or post poliomyelitis or postpolio syndrome or (postpolio and syndrome) or PPS).mp.
11. or/1-10
12. exp Ventilators, Mechanical/
13. respiration, artificial/ or interactive ventilatory support/ or noninvasive ventilation/ or exp positive-pressure respiration/
14. Pulmonary Ventilation/
15. ventilat*.ti. or (negative pressure ventilat* or mechanical ventilat* or positive-pressure ventilat* or (non-invasive adj4 ventilat*) or NIV or NIPPV or VAI).mp.
16. or/12-15
17. home care services/ or home care services, hospital-based/ or home nursing/
18. home.ti. or (home adj6 ventilat*).mp. or (home.mp. and Long-Term Care/) or Home care.mp. or homecare.mp. or HMV.mp. or (assisted living and ventilat*).mp.
19. 17 or 18
20. 11 and 16 and 19
21. limit 20 to yr="1996 -Current"

EBM Reviews - Cochrane Central Register of Controlled Trials July 2013
(Same search as Medline)
Search date: August 20 2013 (39 results)

Embase 1974 to 2013 August 19
(971 results)
1. lung disease/
2. obstructive airway disease/ or chronic obstructive lung disease/
3. neuromuscular disease/
4. exp motor neuron disease/
5. exp muscular dystrophy/
6. myotonic dystrophy/
7. exp spinal cord injury/
8. respiratory failure/ or chronic respiratory failure/
9. exp hypoventilation/
10. breathing disorder/
11. hypercapnia/
12. exp kyphosis/
13. exp scoliosis/
14. poliomyelitis/
15. (COPD or respiratory insufficiency or respiratory failure or breathing failure or breathing difficult* or respiratory muscle weakness or ("pulmonary function" and failure) or pulmonary disease or amyotrophic lateral sclerosis or ALS or OHS or hypoventilation or spinal cord injur* or SCI or muscular dystroph* or Kyphoscoliosis or Postpoliomyelitis Syndrome or post-polio syndrome or post polio syndrome or polio syndrome or post-poliomyelitis or post poliomyelitis or postpolio syndrome or (postpolio and syndrome) or PPS).mp.
16. or/1-15
17. ventilator/ or intermittent positive pressure breathing machine/ or mechanical ventilator/
18. artificial ventilation/ or interactive ventilatory support/ or intermittent mandatory ventilation/ or intermittent positive pressure ventilation/ or noninvasive ventilation/
19. lung ventilation/
20. ventilat*.ti. or (negative pressure ventilat* or mechanical ventilat* or positive-pressure ventilat* or (non-invasive adj4 ventilat*) or NIV or NIPPV or VAI).mp.
21. or/17-20
22. home care/ or home respiratory care/
23. home.ti. or (home adj6 ventilat*).mp. or (home.mp. and Long-Term Care/) or Home care.mp. or homecare.mp. or HMV.mp. or (assisted living and ventilat*).mp.
24. 22 or 23
25. 16 and 21 and 24
26. limit 25 to yr="1996 -Current"

Cochrane Library (including Cochrane Database of Systematic Reviews (CDSR) and Other Reviews (DARE) and Technology Assessments (HTA) and Economic Evaluations (NHS EED)
Searched August 20, 2013 (2 results in NHS EED and 7 results in CDSR)

Search strategy
(chronic obstructive pulmonary disease or COPD or myotonic dystroph* or respiratory insufficiency or respiratory failure or breathing failure or breathing difficult* or respiratory muscle weakness or ("pulmonary function" and failure) or pulmonary disease or amyotrophic lateral sclerosis or ALS or OHS or hypoventilation or spinal cord injur* or SCI or muscular dystroph* or Kyphoscoliosis or Postpoliomyelitis Syndrome or post-polio syndrome or post polio syndrome or polio syndrome or post-poliomyelitis or post poliomyelitis or postpolio syndrome or (postpolio and syndrome) or PPS) *in title abstract keywords*
AND (ventilat* or NIV or NIPPV or VAI) *in title abstract keywords*
AND (home) *in title abstract keywords*

CINAHL PLUS with FULL TEXT
Search August 20, 2013
Results (135)

S19 S11 AND S14 AND S17 *Limiters - Published Date: 19960101-20131231*
S18 S11 AND S14 AND S17
S17 (S15) AND (S15 OR S16)
S16 home.ti. OR ((home n6 ventilat*) or home care or homecare or hmv or (assisted living AND ventilat*))
S15 (MH "Home Health Care+") OR (MH "Home Respiratory Care+") OR (MH "Home Care Equipment and Supplies")
S14 S12 OR S13
S13 TI ventilat* OR ((negative pressure ventilat* or mechanical ventilat* or positive-pressure ventilat* or (non-invasive n4 ventilat*) or NIV or NIPPV or VAI))
S12 (MH "Ventilators, Mechanical") OR (MH "Respiration, Artificial") OR (MH "Positive Pressure Ventilation") OR (MH "Ventilation, Negative Pressure")
S11 S1 OR S2 OR S3 OR S4 OR S5 OR S6 OR S7 OR S8 OR S9 OR S10
S10 COPD or respiratory insufficiency or respiratory failure or breathing failure or breathing difficult* or respiratory muscle weakness or ("pulmonary function" and failure) or pulmonary disease or amyotrophic lateral sclerosis or ALS or OHS or hypoventilation or spinal cord injur* or SCI or muscular dystroph* or Kyphoscoliosis or Postpoliomyelitis Syndrome or post-polio syndrome or post polio syndrome or polio syndrome or post-poliomyelitis or post poliomyelitis or postpolio syndrome or (postpolio and syndrome) or PPS)
S9 (MH "Kyphosis") OR (MH "Scoliosis")
S8 (MH "Spinal Cord Injuries+")
S7 (MH "Hypercapnia")
S6 (MH "Respiration Disorders")
S5 (MH "Hypoventilation+")

S4 (MH "Respiratory Failure")
S3 (MH "Myositis") OR (MH "Myotonic Disorders+")
S2 (MH "Neuromuscular Diseases") OR (MH "Motor Neuron Diseases+") OR (MH "Muscular Disorders, Atrophic") OR (MH "Muscular Atrophy, Spinal")
S1 (MH "Lung Diseases") OR (MH "Pulmonary Disease, Chronic Obstructive") OR (MH "Lung Diseases, Obstructive")

Assessment of Study Quality

Modified Downs and Black Study Quality assessment for observational studies. [3]

Reporting

1) Is the hypothesis/aim/objective of the study clearly described?
2) Are the main outcomes to be measured clearly described in the Introduction or Methods section?
3) Are the characteristics of the patients included in the study clearly described?
4) Are the main findings of the study clearly described?
5) Does the study provide estimates of the random variability in the data for the main outcomes**?**
6) Have adverse events that may be a consequence of the intervention been reported?
7) Have the characteristics of patients lost to follow-up been described?
(A point was given to surveys only if there was a complete response rate)
8) Have actual probability values been reported (e.g. 0.035 rather than <0.05) for the main outcomes except where the probability value is less than 0.001?

External validity

9) Were the subjects asked to participate in the study representative of the entire population from which they were recruited?
(A point was given to surveys only if there was a complete patient response rate and subjects were representative of the entire population)
10) Were the staff, places, and facilities where the patients were treated, representative of the treatment the majority of patients receive?

Internal validity – bias

11) If any of the results of the study were based on "data dredging", was this made clear?
12) Do the analyses adjust for different lengths of follow-up of patients?
13) Were the statistical tests used to assess the main outcomes appropriate?
14) Was compliance with the intervention/s reliable? (≥4 hours/night ≥5 days/week was chosen as this would be considered compliant by most)
15) Were the main outcome measures used accurate (valid and reliable)?.

Internal validity – confounding

16) Was there adequate adjustment for confounding in the analyses from which the main findings were drawn?
17) Were losses of patients to follow-up taken into account?
(A point was given to surveys only if there was a complete response rate)
18) Did the study comment on power?

Disclosure

19) Was funding disclosed?
For grading methodological quality a discussion between 2 authors (E.M. and L.A.) was conducted and consensus reached on how to best modify the Downs and Black scoring system. Each study was rated independently and consensus reached. Scores range from 0 (poor quality) to 19 (rigorous).

SUPPLEMENTARY RESULTS

Categorization of disease states, which lead to CRF and the subsequent establishment of HMV

1) Neuromuscular disorders (NMD): Post-poliomyelitis, Duchenne muscular dystrophy, muscular dystrophies other than Duchenne (Becker and limb girdle), spinal cord injury, myotonic dystrophy, Steinert's disease, myopathies, spinal muscular atrophy (type I-III), motor neuron disease (includes amyotrophic lateral sclerosis), spina bifida, polyneuropathy, Charcot-marie- Tooth disease, congenital hypoventilations syndrome, phrenic neuropathy/injury, Guillain-Barre syndrome, multiple sclerosis, Freidreich's ataxia, Werdnig-Hoffman disease polymysositis/dermatomyositis, post cerebral accident
2) Restrictive thoracic diseases (RTD) or Chest wall diseases (CWD) – post tuberculosis sequela, kyphoscoliosis, scoliosis, pectus excavatum, pleural fibrosis
3) Chronic obstructive disease (COPD) – cystic fibrosis, lung parenchymal, bronchiectasis, pulmonary fibrosis
4) Obesity hypoventilation syndrome (OHS) – overlap syndrome
5) Miscellaneous- complex congenital pulmonary diseases, ankylosing spondylitis, upper airway deformity, tracheomalacia,, cystic fibrosis, birth injury, burns

Slight variations in disease categorization across studies did occur.

Supplementary Table 1. Modified Downs and Black score by category across studies

Study	Reporting (8)	External validity (2)	Bias (5)	Confounding (3)	Disclosure (1)	Total (19)
Bourke et al[4]	7	2	5	3	1	18
Tsolaki et al[5]	6	2	5	2	0	15
Windish et al[6]	7	2	5	2	0	16
Dellborg et al[7]	6	2	4	0	1	13
Farrero et al[8]	8	2	5	1	0	16
Gonzalez et al[9]	6	0	2	0	0	8
Janssens et al[10]	7	2	5	1	0	15
Nauffal et al[11]	5	2	4	1	0	12
Hein et al[12]	7	2	3	1	0	13
Bach et al[13]	6	2	4	1	0	13
Janssens et al[14]	7	2	4	1	0	14
Chatwin et al[15]	7	2	4	1	0	14
Budweiser et al[16]	7	2	5	2	1	17
Marchese et al[17]	7	2	5	2	1	17
Evans et al[18]	6	1	4	0	1	12
Chang et al[19]	6	1	3	0	0	10
Fernandez-Alvarez et al[20]	6	0	3	1	0	10
Lopez-Campos et al[21]	7	1	4	1	1	15
Vitacca et al[22]	6	2	4	1	0	13
Tsara et al[23]	5	2	3	0	0	10
Kaub-Wittemer et al.[24]	3	0	2	0	0	5
Markstrom et al[25]	5	1	3	0	0	9
Van Kesteren et al{vanKesteren:2001uc}	4	1	3	0	0	8
Sevick et al[26]	5	1	3	0	0	9
Moss et al[27]	2	1	3	0	0	6
Botel et al[28]	3	0	1	0	0	4
Mean score	5.9	1.4	3.7	0.8	0.2	12
Median score	6	2	4	1	0	13

Health Related Quality of Life Instruments[29]

Generic

1. Medical outcomes study short form survey (MOS-SF 36): generic instrument comprised of 36 items grouped within the domains of physical functioning, physical role, emotional role, bodily pain, vitality, social functioning, mental health and general health. These 8 categories can be further grouped into the following summary measures: physical capacity summary and mental capacity summary.

2. Sickness Impact Profile (SIP): a generic measure used to evaluate the impact of disease on both physical and emotional functioning. It includes 136 items

grouped by physical domains (ambulation, mobility, body care and movement) and psychosocial domains (social interaction, communication, alertness behavior, emotional behavior; sleep and rest, eating, home management, recreation and pastimes, employment) Scores range from 0-100%, with higher scores correlating with more serious dysfunction. A score of >10% indicates clinical handicap.

3. Profile of mood states (POEMS): 65 adjectives that are rated by the individual on a 5 point scale that form the following 6 factors: tension-anxiety, depression-dejection, anger-hostility, fatigue-inertia, vigor-activity and confusion-bewilderment.

4. Munich Quality of Life Dimensions (MLDL): Scores general satisfaction on a 1 (not satisfied) to 10 (very satisfied) scale.

5. Health Index (HI): Comprised of the following 11 items: fatigue, energy, sleep, mobility, mood, loneliness, bowel function, vertigo, pain, health during the past week and general health compared to other people. Score is rated 1 (very poor) to 4 (very good)

6. Sense of coherence scale (SOC): 13 items are summarized to form an overall score. The higher the score the stronger the sense of coherence and the better the perceived health.

Disease specific

7. Saint George respiratory questionnaire (SGRQ): Designed to measure impact on overall health, daily life and perceived well-being in patients with fixed and reversible airway obstruction. 76 items grouped by symptoms (frequency and severity) rated on a 5-point Likert scale, Activity (activities that are limited by breathlessness) and Impacts (social function and psychological disturbances) rated as dichotomous (yes/no). Higher scores indicate poor health.
8. Chronic Respiratory Disease Questionnaire (CRQ): 20 item questionnaire rated on a 7-point modified Likert scale and grouped across the following 4 domains: dyspnea, fatigue, emotional function and mastery. Higher score indicate better QOL

9. Severe Respiratory Insufficiency (SRI) Questionnaire: Developed for use by patients with respiratory failure receiving mechanical ventilation. Includes 49 items rated on a 5 point Likert scale and grouped by the following domains: Respiratory complaints, physical functioning, attendant symptoms and sleep, social relationships, anxiety, psychological well-being and social functioning.

Caregiver QOL assessment tools[30-32]

1) Caregiver burden inventory: Developed in caregivers of confused or disoriented older persons. It addresses the following 5 common areas of burden: time-dependent burden (due to the restriction of time), physical burden (feelings of fatigue and chronic health problems), social burden (relationships with family members and friends), emotional burden (negative or positive feelings toward the patient), and developmental burden (the perception of being excluded from the expectations and opportunities enjoyed by one's peers).

2) Zarit interview: Developed to assess burden for caregivers of patients with senile dementia in 1980. Originally contained 29 items, but the revised version contains 22 items rated on a 5-point Likert scale. They attempted to cover the areas of caregiver health, psychological well being, finances, social life and relationship between the caregiver and the patient in order to form the following 2 models: personal strain and role strain. Variations exist.*)*

3) Family Burden Questionnaire: Developed to assess burden on family members of patients with depression. 95 items measured on a 3-level scale, which address burden in the areas of employment, household management, finances and social relations. The questionnaire examines both objective (the buden which the caregiver identified to exist as a consequence of their situation) and subjective (the burden which the caregivers felt to influence their lifestyle).

References

1 Stroup DF, Berlin JA, Morton SC, *et al.* Meta-analysis of Observational Studies in Epidemiology. *JAMA* 2000;**283**:2008–12.

2 2011 TCC. Cochrane Handbook for Systematic Reviews of Interventions. *Available from wwwcochrane-handbookorg*

3 Downs SH, Black N. The feasibility of creating a checklist for the assessment of the methodological quality both of randomised and non-randomised studies of health care interventions. *Epidemiol Community Health* 1998;**52**:377–84.

4 Bourke SC, Tomlinson M, Williams TL, *et al.* Effects of non-invasive ventilation on survival and quality of life in patients with amyotrophic lateral sclerosis: a randomised controlled trial. *Lancet Neurol* 2006;**5**:140–7. doi:10.1016/S1474-4422(05)

5 Tsolaki V, Pastaka C, Kostikas K, *et al.* Noninvasive Ventilation in Chronic Respiratory Failure: Effects on Quality of Life. *Respiration* 2011;**81**:402–10. doi:10.1159/000317138

6 Windisch W, on behalf of the quality of life in home mechanical ventilation study group. Impact of home mechanical ventilation on health-related quality of life. *European Respiratory Journal* 2008;**32**:1328–36. doi:10.1183/09031936.00066407

7 Dellborg C, Olofson J, Midgren B, *et al.* Impact of home mechanical ventilation on health-related quality of life in patients with chronic alveolar hypoventilation: a prospective study. *The Clinical Respiratory Journal* 2007;**2**:26–35. doi:10.1111/j.1752-699X.2007.00034.x

8 Farrero E, Prats E, Manresa F, *et al.* Outcome of non-invasive domiciliary ventilation in elderly patients. *Respiratory Medicine* 2007;**101**:1068–73. doi:10.1016/j.rmed.2006.10.005

9 Gonzalez C, Ferris G, Diaz J, *et al.* Kyphoscoliotic Ventilatory Insufficiency. *Chest Journal* 2003;**124**:857–62.

10 Janssens JP, Derivat S, Breitenstein E, *et al.* Changing Patterns in Long-term Noninvasive Ventilation. *Chest Journal* 2003;**123**:67–79.

11 Nauffal D, Doménech-Clar R, Martinez-Garcia MA, *et al.* Noninvasive positive pressure home ventilation in restrictive disorders: outcome and impact on health-related quality of life. *Respiratory Medicine* 2002;**96**:777–83. doi:10.1053/rmed.2002.1347

12 Hein H, Schucher B, Magnussen H. Quality of life of various patient groups

during home mechanical ventilation. *Med Klin (Munich)* 1999;**94**:99–101.

13 Bach JR, Rajaraman R, Ballanger F, *et al.* Neuromuscular Ventilatory Insufficiency. Effect of Home Mechanical Ventilator Use v Oxygen Therapy on Pneumonia and Hospitalization Rates. *Med Rehabil* 1998;**77**:8–19.

14 Janssens JP, Cicotti E, Fitting JW, *et al.* Non-invasive home ventilation in patients over 75 years of age: tolerance, compliance, and impact on quality of life. *Respiratory Medicine* 1998;**92**:1311–20.

15 Chatwin M, Heather S, Hanak A, *et al.* Analysis of home support and ventilator malfunction in 1,211 ventilator-dependent patients. *European Respiratory Journal* 2010;**35**:310–6. doi:10.1183/09031936.00073409

16 Budweiser S, Hitzl AP, Jörres RA, *et al.* Health-related quality of life and long-term prognosis in chronic hypercapnic respiratory failure: a prospective survival analysis. *Respir Res*;**8**:92.

17 Marchese S, Coco Lo D, Coco Lo A. Outcome and attitudes toward home tracheostomy ventilation of consecutive patients: A 10-year experience. *Respiratory Medicine* 2008;**102**:430–6. doi:10.1016/j.rmed.2007.10.006

18 Evans R, Catapano M, Brooks D, *et al.* Family caregivers perspectives on caring for ventilator-assisted individuals at home. *Canadian Respiratory journal* 2012;**19**:1–373–379.

19 Chang AY, Marsh S, Smith N, *et al.* Long-term community non-invasive ventilation. *Internal Medicine Journal* 2010;**40**:764–71. doi:10.1111/j.1445-5994.2010.02171.x

20 Fernandez-Alvarez R, Rubinos-Cuadrad G, Cabrera-Lacalzada C, *et al.* Home Mechanical Ventilation: Dependency and Burden of Care in the Home. *Arch Bronconeumol* 2009;**45**:383–6.

21 López-Campos JL, Failde I, Masa JF, *et al.* Factors related to quality of life in patients receiving home mechanical ventilation. *Respiratory Medicine* 2008;**102**:605–12. doi:10.1016/j.rmed.2007.11.005

22 Vitacca M, Assoni G, Pizzocaro P, *et al.* A pilot study of nurse-led, home monitoring for patients with chronic respiratory failure and with mechanical ventilation assistance. *j telemed telecare* 2006;**12**:337–42. doi:10.1258/135763306778682404

23 Tsara V, Serasli E, Voutsas V, *et al.* Burden and Coping Strategies in Families of Patients under Noninvasive Home Mechanical Ventilation. *Respiration* 2006;**73**:61–7. doi:10.1159/000087460

24 Kaub-Wittemer D, Steinbüchel NV, Wasner M, *et al.* Quality of life and psychosocial issues in ventilated patients with amyotrophic lateral sclerosis and their caregivers. *Journal of Pain and Symptom Management* 2003;**26**:890–6. doi:10.1016/S0885-3924(03)00323-3

25 Markstrom A, Sundell K, Lysdahl M, *et al.* Quality-of-Life Evaluation of Patients With Neuromuscular and Skeletal Diseases Treated With Noninvasive and Invasive Home Mechanical Ventilation. *Chest Journal* 2002;**122**:1695–700.

26 Sevick MA, Bradham DD. Economic value of caregiver effort in maintaining long-term ventilator-assisted individuals at home. *Heart & Lung* 1997;**26**:148–57.

27 Moss AH, Oppenheimer EA, Casey P, *et al.* Patients with Amyotrophic Lateral Sclerosis Receiving Long-term Mechanical Ventilation. *Chest Journal* 1996;**110**:249–55.

28 Botel U, Glaser E, Niedeggen A, *et al.* The cost of ventilator-dependent spinal cord injuries-patients in the hospital and at home. *Spinal Cord* 1997;**35**:40–2.

29 AMERICAN THORACIC SOCIETY. Quality of Life Resource. Last updated 2008. Retrieved Oct 4,2014 from http://qol.thoracic.org/sections/instruments/index.html. 1999.

30 Novak M, Guest C. Application of a Multidimensional Caregiver Burden Inventory. *The Gerontologist* 2008;**29**:798–803.

31 Schene AH, Tessler RC, Gamache GM. Instruments measuring family or caregiver burden in severe mental illness. *Soc Psychiatry Psychiatr Epidemiol* 1994;**29**:228–40.

32 Zarit SH, Reever KE, Bach-Peterson J. Relatives of the Impaired Elderly: Correlates of Feelings of Burden. *The Gerontologist* 1980;**20**:649–55.
